# Multienzymatic Platform for Coupling a CCU Strategy to Waste Valorization: CO_2_ from the Iron and Steel Industry and Crude Glycerol from Biodiesel Production

**DOI:** 10.1021/acssuschemeng.4c04908

**Published:** 2025-01-17

**Authors:** Sady Roberto Rodriguez, Gregorio Álvaro, Marina Guillén, Oscar Romero

**Affiliations:** Bioprocess Engineering and Applied Biocatalysis Group, Department of Chemical, Biological and Environmental Engineering, Universitat Autònoma de Barcelona, 08193 Bellaterra, Spain

**Keywords:** CO_2_ reduction, carbon capture and utilization, waste valorization, multienzymatic system, high-value chemicals

## Abstract

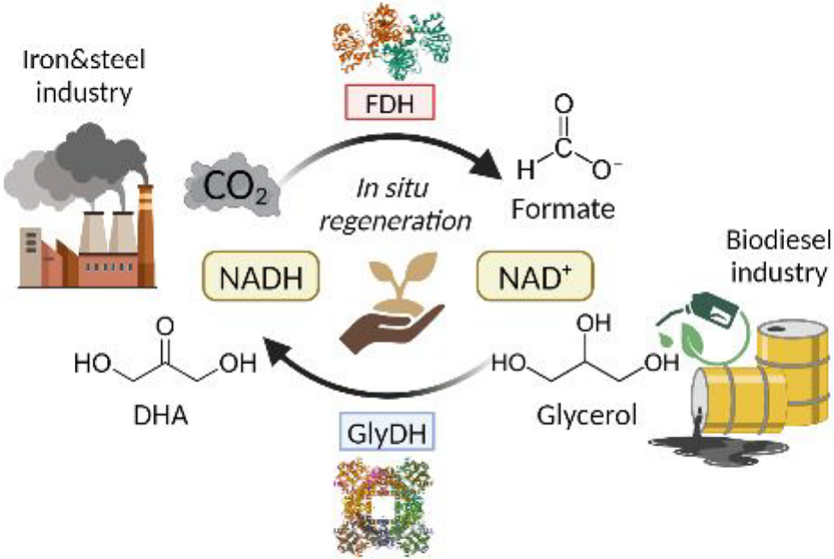

Ongoing climate crisis demands the development of carbon capture and utilization (CCU) technologies that emphasize simplicity, eco-sustainability, and cost-effectiveness. Enzymatic CO_2_ reduction emerges as an alternative to biotransforming this cheap raw material into high-value products under milder conditions. This work proposes a multienzymatic platform to reduce CO_2_ to formate by formate dehydrogenase (FDH) and oxidize glycerol to dihydroxyacetone (DHA) by glycerol dehydrogenase (GlyDH), allowing for efficient cofactor regeneration. Through studies such as pH operating range, enzyme stability, FDH/GlyDH ratio, and reaction medium engineering to achieve optimal soluble CO_2_ concentrations, the reaction with a gas mixture of 24% CO_2_ yielded 5.7 mM formate and 6 mM DHA after 30 h, achieving a 92.3% CO_2_ conversion. To evaluate the feasibility under industrially relevant conditions, a synthetic gas mixture mimicking the composition of the iron and steel industry off-gases (24.5% CO_2_) and crude glycerol (64% v/v) from biodiesel production was tested as substrates. The simultaneous production was successful, yielding 3.1 mM formate and 4.4 mM DHA. Formic acid was subsequently purified using liquid–liquid extraction, employing the green solvent 2-methyltetrahydrofuran (2-MTHF). For the first time to our knowledge, a CCU strategy has been successfully coupled with industrial waste valorization, obtaining two high-value molecules by means of a robust, profitable, and easily manageable multienzymatic system.

## Abstract 2

Capturing CO_2_ from industrial streams is feasible for achieving carbon neutrality and producing high-value chemicals through a multienzymatic strategy.

## Introduction

Global greenhouse gas emissions from industrial activities continue to rise, mainly from sectors, such as electricity generation, construction, and transportation. The International Energy Agency has proposed to increase the CO_2_ capture and utilization.^1^ Therefore, to attain the goal of “carbon neutrality”, it is essential to foster the development of innovative carbon capture and utilization (CCU) technologies that prioritize simplicity, cost-effectiveness, and rigorous regulatory oversight. With CCU methodologies, it becomes feasible to convert a cheap feedstock like CO_2_ into high-value products at reduced costs and energy consumption and also with diminished fossil carbon dependence.^2^

The iron and steel industry (ISI) is the sector with the highest energy consumption on the planet, accounting for 23% of the total global energy consumption while concurrently contributing to 28% of the overall CO_2_ emissions by industries.^3^ The demand for iron and steel is expected to increase in the coming years.^4^ Hence, it is necessary to develop low-carbon alternatives capable of diminishing CO_2_ emissions while concurrently meeting the escalating global demand for iron and steel.

Enzymatic CO_2_ biotransformation is of high interest since reduction reaction products, such as formic acid, formaldehyde, and methanol, are characterized by having numerous industrial applications. Enzymes like formate dehydrogenase (FDH) exhibit a pronounced affinity for CO_2_ while avoiding interaction with other dissolved species such as bicarbonate and carbonic acid.^5^ FDH catalyzes the conversion of CO_2_ to formate through an oxidation–reduction reaction with NADH consumption. This biocatalyst has been considered as inspiration and guidance on methods for CO_2_ fixation, which is relevant for addressing global warming.^6^ The FDH from *Candida boidinii* stands out as the most representative metal-dependent variant due to its commercial availability and wide-ranging industrial applications.^7^

One of the pivotal factors in enzymatic CO_2_ reduction is the electron donor. NADH is by far the most common electron donor for all types of FDH. However, its high cost represents a bottleneck for this system. To solve this issue, in situ cofactor regeneration is mandatory. In terms of cost-effectiveness, enzymatic cofactor regeneration emerges as a more favorable alternative over electrochemical and photochemical systems,^8,9^ characterized by its simplicity, eco-friendliness, and for being a well-established technology. Some cofactor regeneration enzyme schemes have been explored with different sacrificial substrates, such as glycerol, phosphite, glucose, and glutamine.^10^ Glycerol is considered as an eco-friendly and low-cost resource with substantial opportunities for reassessment within the industry. The biodiesel industry produces large quantities of glycerol waste through the transesterification process. About 10% of crude glycerol is generated as a byproduct.^11^ However, the management of this waste poses a significant environmental challenge, and its purification represents high costs.^12^ Therefore, it is necessary to develop alternatives for bioconverting this molecule since it is widely available but barely manageable.

**Scheme 1:**
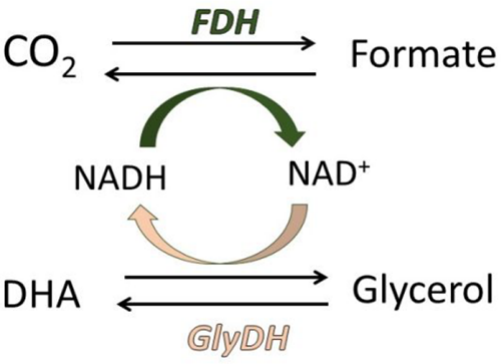
Multienzymatic System for the Coproduction of Formate from CO_2_ and DHA from Glycerol, Featuring In Situ NADH Regeneration The system comprises formate dehydrogenase (FDH) and glycerol dehydrogenase (GlyDH) enzymes.

In this study, we report a proof of concept for a CCU strategy based on a one-pot multienzymatic system to produce formate, a compound with a global market value of approximately $US 655 million^13^ and applications in the pharmaceutical, food, and textile industries. To facilitate cofactor regeneration, glycerol is oxidized by the glycerol dehydrogenase (GlyDH) enzyme to produce dihydroxyacetone (DHA), and concurrently, NAD^+^ is reduced back to its NADH form (Scheme 1). DHA is recognized as a building block in the pharmaceutical and cosmetic industries, with a global market value of approximately $US 171.7 million.^14^ Thus, the proposed multienzymatic platform, based on the circular economy principles, will allow coupling a CCU strategy with waste valorization, producing two compounds of industrial interest.

The developed system will be finally assessed under an industrially relevant environment using a synthetic gas mixture, mimicking real blast furnace off-gas composition from the iron and steel industry and real crude glycerol from industrial biodiesel production. For the first time to our knowledge, the biotransformation of CO_2_ and industrial waste into high-added-value molecules—formate and DHA—is achieved through a multienzymatic system, employing CCU methodology, soluble enzymes, and cofactor regeneration.

## Experimental Section

### Materials

All reagents were purchased from Sigma-Aldrich (St. Louis, MO, USA) and PanReac Quimica S.L.U. (Barcelona, Spain). The cofactors NADH and NAD^+^ were purchased from GERBU Biotechnik GmbH (Heidelberg, Germany). All samples and buffers were prepared in Milli Q water (18.2 MΩ·cm, Merck Millipore, USA). A gas mixture with 24% CO_2_ and 76% N_2_ and a gas mixture mimicking real blast furnace off-gas composition (24.5% CO_2_, 46.6% N_2_, 23.9% CO, 1.2% O_2_, and 3.8% H_2_)^15^ were obtained from Carburos Metalicos (Barcelona, Spain). The crude glycerol (glycerol 64% v/v; full composition in the Supporting Information [SI]) was kindly provided by ecoMotion Biodiesel S.A. (Barcelona, Spain). Formate dehydrogenase (EC 1.17.1.9) and glycerol dehydrogenase (EC 1.1.1.6) enzymes were produced and purified by the research group according to the procedures found in the SI. Specific activities were 11.8 and 6.3 U/mg for FDH and GlyDH, respectively.

### Catalytic Activity Tests

FDH and GlyDH activities were determined based on the reduction rate of NAD^+^ to NADH induced by the oxidation of formate to CO_2_ and glycerol to DHA, respectively. For the FHD activity assay, 100 mM phosphate buffer (pH 7.5), 50 mM sodium formate, and 1.67 mM NAD^+^ were employed. In the case of GlyDH, 100 mM glycerol dissolved in 100 mM phosphate buffer (pH 7.0) and 2.5 mM NAD^+^ were used. The assays were performed in a SPECTROstar microplate reader at 340 nm and incubated at 30 °C. One unit of FDH/GlyDH activity corresponds to the formation of 1 μmol of NADH per minute under these conditions.

### Optimum pH and Stability of Enzymes

The optimal pH of each enzyme was determined by changing the pH of the activity assay and performing the corresponding assay for each enzyme. The enzymes were incubated in a Britton–Robinson buffer—50 mM acetic acid, phosphoric acid, and boric acid—and adjusted with NaOH to obtain solutions in a pH range of 6–10.

The stability of the enzymes was evaluated by incubating the samples under the same pH conditions (from 6 to 10), at a temperature of 30 °C, and shaking at 450 rpm in a MultiTherm heat block system (Benchmark Scientific Inc.). These experiments were conducted in a reaction volume of 15 mL. Samples were taken at different times to measure the catalytic activity of each enzyme by means of the activity tests previously described. Triplicate assays were performed.

### Reaction Medium Engineering

Phosphate, Britton–Robinson, Tris-HCl, MOPS, HEPES, Gly–Gly, and MES buffers, all at concentrations of 250 mM, were chosen as potential candidates for the reaction medium. The concentration of soluble CO_2_ was monitored using a CO_2_ sensor InPro5000*i*/12/220 (Metter Toledo S.A.E., Barcelona, Spain). The formate production was carried out in each of these reaction media, which was previously saturated with pure CO_2_. The reaction volume for these experiments was 15 mL. Subsequently, the initial pH of the reaction was measured. In addition, 10 mM NADH and 0.5 U·mL^–1^ purified FDH enzyme were employed to perform the reaction for 24 h at a temperature of 30 °C and constant stirring at 450 rpm. Triplicate assays were performed.

### Multienzymatic Synthesis of Formate and DHA with NADH Regeneration

Formate and DHA coproduction was conducted using 250 mM phosphate buffer, previously saturated with CO_2_ from the gas sources (gas mixture with 24% CO_2_ or synthetic gas mixture) at a flow rate of 30 mL·min^–1^ until a constant CO_2_ concentration was achieved. The initial pH for the reaction was measured. 100 mM glycerol (pure and crude) and 1 mM NADH were used. Controls of each substrate were evaluated, as well as a mixture of CO_2_ (presaturated in the medium) and glycerol. A range of ratios of purified FDH/GlyDH enzymes was employed, considering an FDH concentration of 0.5 U·mL^–1^. The reaction was carried out in a reaction volume of 12 mL for 30 h at a temperature of 30 °C and continuously stirred at 450 rpm using the MultiTherm Heat Block system (Benchmark Scientific Inc.). Catalytic activity of both enzymes was also measured throughout the reaction. Likewise, controls of both enzymes were studied under nonreactive conditions. Triplicate assays were performed.

### Stability Study of Enzymes under Relevant Industrial Environments: Synthetic Gas Mixture and Real Crude Glycerol

The stability of the enzymes was evaluated by incubating each enzyme in 250 mM phosphate buffer, previously saturated with 320 mg·L^–1^ CO_2_ from the synthetic gas mixture and real crude at a 100 mM glycerol concentration. The reaction was carried out in a reaction volume of 12 mL for 30 h at a temperature of 30 °C and constant stirring at 450 rpm. The relative activity was calculated considering zero-time samples as 100% catalytic activity.

### Formate Isolation from the Reaction Medium

The formate was initially isolated by a liquid–liquid extraction method with an organic solvent 2-methyltetrahydrofuran (2-MTHF). The reaction medium was acidified to a pH of 2–4 using 6 M HCl to protonate the formate. Following this, four successive extractions were carried out with 2-MTHF at a 1:1 volume ratio in a separation funnel, with vigorous shaking for 5 min per extraction. The formic acid extracted into the 2-MTHF phase was then separated by distillation using a Heidolph WB 2000 rotary evaporator at 30 °C for 25 min. Formic acid obtained from distillation was resuspended in water for quantification.

### HPLC Analysis

Formate, DHA, and glycerol quantification were performed in an Agilent 1220 Infinity II liquid chromatograph using an ion exchange method, with the IC-Sep COREGEL 87H3 column and 0.5 mM sulfuric acid (H_2_SO_4_)/acetonitrile (65:35) as the mobile phase. A flow rate of 0.6 mL·min^–1^, an injection volume of 20 μL, a column temperature of 30 °C, a UV/visible detector at 210 nm, and an RID detector at 30 °C were used for this analysis. For glycerol 1,2-carbonate quantification, a reverse phase method was used with the C-18 CORTECS column and 0.004 N sulfuric acid (H_2_SO_4_) as the mobile phase. A flow rate of 0.8 mL·min^–1^, an injection volume of 100 μL, and a column temperature and an RID detector at 35 °C were used for this analysis. Formic acid in 2-MTHF was quantified using a C-18 CORTECS column for its analysis in an organic phase, with 0.5 mM sulfuric acid (H_2_SO_4_)/acetonitrile (65:35) as the mobile phase. A flow rate of 0.6 mL·min^–1^, an injection volume of 20 μL, a column temperature of 30 °C, and a UV/visible detector at 210 nm were used. Triplicate assays were performed. Chromatograms and retention time are shown in the SI (Figures S7–S9).

## Results and Discussion

### Characterization of FDH and GlyDH Enzymes

The pH level of the medium where enzymatic reactions take place is important to maintain the catalytic efficacy of enzymes. Nevertheless, in this reaction, the concentration of soluble CO_2_ plays a pivotal role in determining the pH of the medium. CO_2_ (g) reacts with water producing carbonic acid, which decreases the pH of the medium. In this context, CO_2_ exists in equilibrium with other chemical species, such as bicarbonate (HCO_3_^–^) and carbonate (CO_3_^2–^). At neutral pH, CO_2_ predominantly exists in equilibrium with bicarbonate.

**Figure 1:**
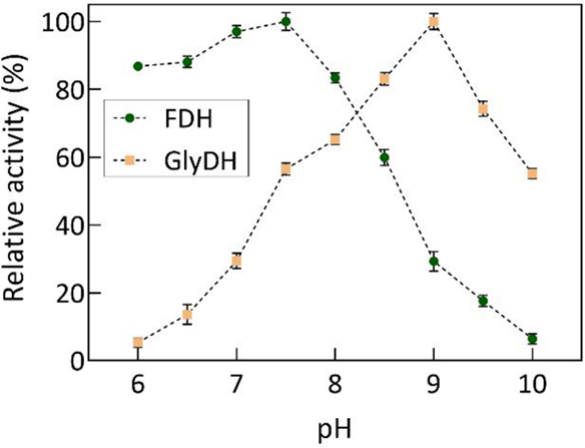
Optimum pH of the FDH and GlyDH enzymes in the pH range of 6–10. The tests were conducted by incubating the samples in a 50 mM Britton–Robinson buffer adjusted to the different pH and at a temperature of 30 °C. The relative activity was calculated by considering as 100% pH, where the enzyme presents the higher catalytic activity.

The determination of the optimal pH for FDH and GlyDH enzymes, and consequently an operational window for the reaction, was conducted by measuring their respective catalytic activities over a pH range of 6–10. As observed in Figure 1, FDH attains its maximum enzymatic activity at pH 7.5, with a pronounced loss of activity at higher pH values (pH ≥ 9.0). Conversely, GlyDH exhibits heightened activity to elevated pH levels (pH ≥ 8.5), achieving its peak activity at pH 9.0, but demonstrates lower activity in the range from 6.0 to 7.0.

As a result, the operational pH window for this enzymatic system was defined within the range from 7.0 to 8.0. At this pH range, GlyDH shows around 30–60% of its total activity according to its pH profile. However, soluble CO_2_ concentrations from 25 to 350 mg·L^–1^ can be achieved within this pH window by using pure CO_2_ (Figure S2).

**Figure 2:**
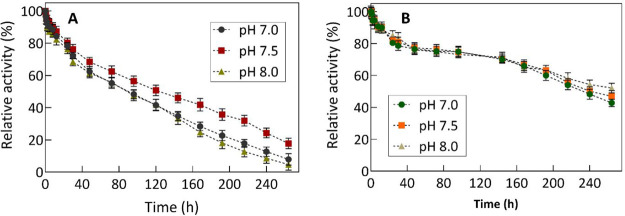
Stability study of the FDH and GlyDH enzymes over time by incubation in a medium with pH corresponding to the operating range of the multienzymatic system (pH 7.0, 7.5, and 8.0). The samples were incubated in a 50 mM Britton–Robinson buffer adjusted to the different pH, at a temperature of 30 °C, with constant stirring at 450 rpm and for 264 h. The relative activity was calculated considering zero-time samples as 100% catalytic activity. (A) FDH and (B) GlyDH.

Following these conditions, the stability over time was evaluated. Both enzymes were incubated in a medium at each pH of the system’s operating range. The maximum stability of FDH is observed at pH 7.5. As a result, an average half-life (*t*_1/2_) of 96 h was obtained under these pH range conditions (Figure 2A). Other FDH variants showed *t*_1/2_ of 61 and 66 h at 30 °C.^16,17^ Some studies have reported shorter half-lives (less than 38 h) at 50 °C^18,19^ and under reactive conditions.^20^ The FDH stability within the pH range of neutrality and slight alkalinity (6.5–9.5) favors its catalytic capacity for formate ion oxidation.^21^ Nevertheless, Choe et al. have substantiated that its stability at neutral pH also ensures effective CO_2_ reduction.^22^

The GlyDH enzyme exhibits greater stability compared to FDH under the examined conditions (Figure 2B). GlyDH from *Geobacillus stearothermophilus* is characterized by being a fairly stable biocatalyst, especially for its thermal stability.^23^ After 96 h, GlyDH still retains more than 74% of its activity, achieving an average *t*_1/2_ of 240 h under these conditions. However, it has been reported that under extreme reaction conditions of pH and temperature, the *t*_1/2_ can be significantly reduced.^24−26^ Hence, the exceptional stability of both soluble enzymes within their operational windows renders them robust biocatalysts suitable for deployment in CCU systems.

### Reaction Medium Engineering

In order to select the most suitable reaction medium, an assessment of various buffers (phosphate, Britton–Robinson, Tris-HCl, MOPS, HEPES, Gly–Gly, and MES) was conducted, guided by four main criteria: (1) The quantity of dissolved CO_2_, (2) the synthesis of formate from dissolved CO_2_ by FDH, (3) NADH, and (4) FDH stability in the medium. All the reaction media evaluated have the capacity to buffer in the pH range from 7.0 to 8.0, which corresponds to the operational window of the multienzymatic system and facilitates the attainment of optimal soluble CO_2_ concentrations.

**Figure 3:**
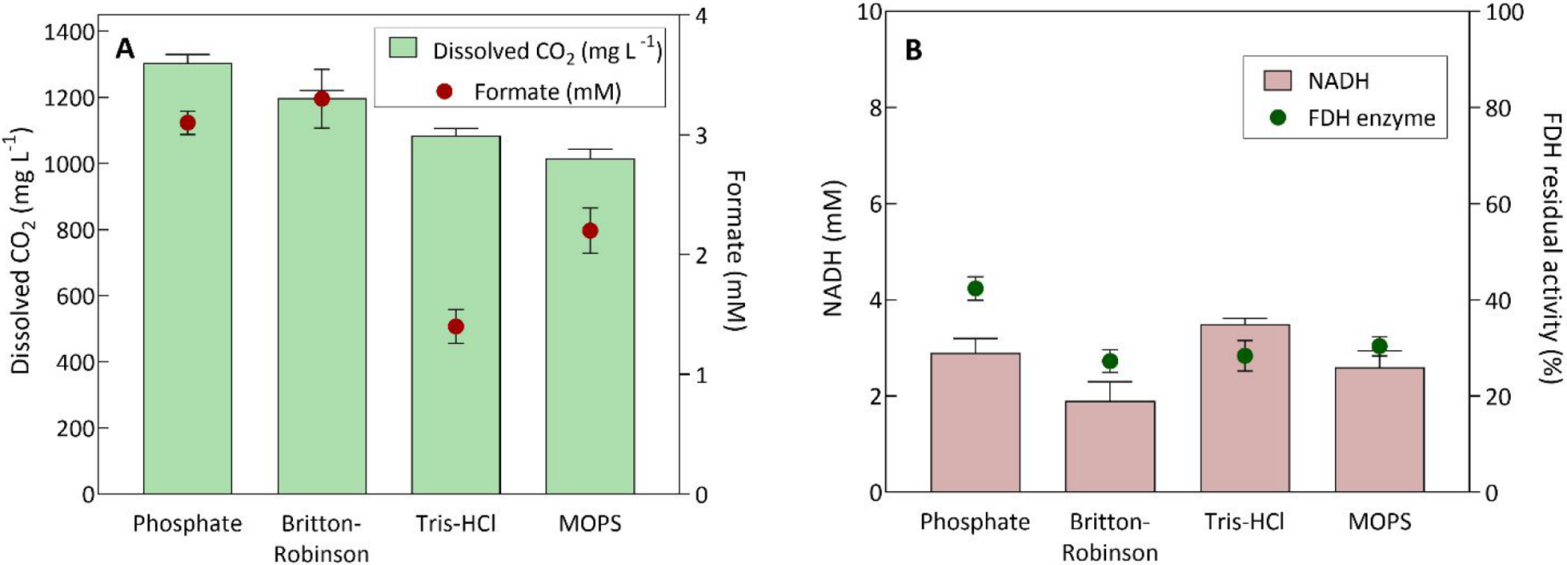
Reaction medium engineering for the multienzymatic system by evaluation of different buffers. The following parameters were considered: (A) Achieved soluble CO_2_ concentration and synthesis of formate and (B) NADH and FDH stabilities in the reaction medium. The formate synthesis reaction was conducted in the buffers at a concentration of 250 mM, previously saturated with pure CO_2_, a reaction volume of 15 mL, initial pH ranges between 6.6 and 6.7, a soluble FDH of 0.5 U·mL^–1^, 10 mM NADH, a temperature of 30 °C, and for 24 h with constant stirring at 450 rpm.

As no formate production was detected using HEPES, Gly–Gly, and MES buffers, they were discarded for further analysis. Regarding the rest of the studied buffer, the highest soluble CO_2_ concentration was reached in the phosphate buffer at 1305 mg·L^–1^ (Figure 3A). This is a satisfactory value compared to CO_2_ solubility in water under standard conditions (1496 mg·L^–1^).^27^ Moreover, a formate production of 3.1 ± 0.13 mM was attained in this medium, marginally trailing the Britton–Robinson buffer (3.3 ± 0.11 mM). Other reaction media such as Tris-HCl and MOPS were also capable of solubilizing >1000 mg·L^–1^ CO_2_ but produced lower yields of formate. As already mentioned, the selection of the media was also based on NADH and FDH stability in the reaction. In the case of NADH, its degradation may be related to its photosensitivity and high instability under acidic conditions.^28^ The stability studies under different pH showed that below pH 7.0, NADH can undergo at least 50.3 ± 2.2% degradation after 24 h. The stability of this molecule is enhanced as the pH increases to the basic region. Above pH 8.5, the NADH stability remains above 50.7 ± 2.1% after 96 h of incubation (Figure S3). As can be seen in Figure 3B, the reaction with phosphate buffer resulted in the lowest percentage of NADH degradation (40 ± 0.89%) at the end of the reaction. Regarding enzyme stability, the FDH showed greater stability in the phosphate buffer, retaining 42.4 ± 0.65% of its activity compared to the residual activity of less than 30% in the other reaction media.

Hence, the phosphate buffer represents a suitable reaction medium for conducting multienzymatic reactions within the system, primarily attributed to its capacity to solubilize CO_2_ available to be used in enzymatic reactions without requiring elevated temperatures, high pressures, or chemical transformation.

In the context of formate synthesis from CO_2_, it is quite interesting to mention that only a few reports using FDH in its soluble form have been documented. Pietricola et al. reported the production of 3.7 mM formate at 2 h using FDH immobilized in natural zeolite but working with a high NADH concentration (14 mM).^29^ Another study reported the production of 2 mM formate within 30 min with FDH immobilized on carbon nanotubes; however, a mixture of bicarbonate/CO_2_ was used as substrates.^30^ The field of electrochemistry has been extensively investigated for the production of formate from CO_2_. Kim successfully demonstrated the production of 3.0 mM formate in a system with NADH regeneration, employing a Cu nanorod/glassy carbon electrode.^31^ On the other hand, Addo examined the bioelectrocatalytic reduction of CO_2_, resulting in a production of 0.7 mM formate within a multienzymatic cascade aimed at methanol production.^32^ Consequently, the soluble FDH enzyme exhibited outstanding performance under mild conditions without the need for complementary immobilization procedures or high enzyme or cofactor concentrations to facilitate CO_2_ reduction.

### Simultaneous Production of Formate and DHA by a Multienzymatic Platform with NADH In Situ Regeneration

**Figure 4:**
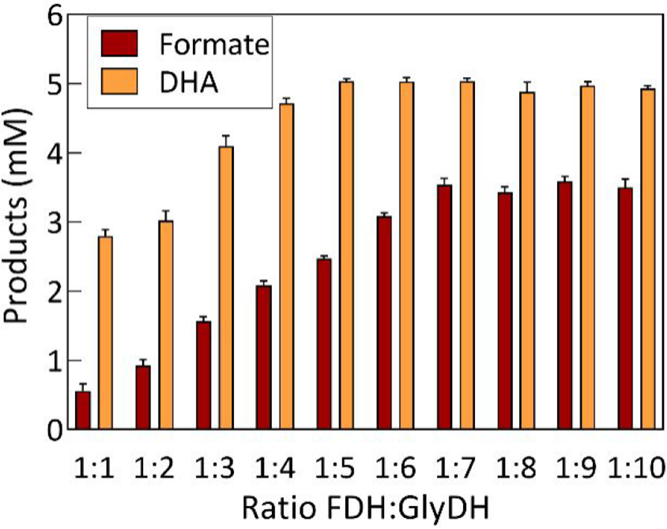
Coproduction of formate and DHA through a multienzyme system with NADH regeneration evaluating different ratios of FDH/GlyDH enzymes. The FDH concentration was 0.5 U·mL^–1^ in all of the assays. The experiments were conducted with 250 mM phosphate buffer as reaction medium, 100 mM glycerol, a gas mixture with 24% CO_2_ (310 mg·L^–1^), and 1 mM NADH. The reactions were carried out at 30 °C, a reaction volume of 12 mL, pH 7.15 (following CO_2_ bubbling), with constant stirring at 450 rpm for 30 h.

Formate and DHA coproduction was conducted on a multienzyme platform featuring NADH cofactor regeneration. First, the determination of the enzyme quantity in the reaction was undertaken by assessing various ratios of FDH/GlyDH (Figure 4). In all experiments, a concentration of 0.5 U·mL^–1^ FDH was applied for the reduction of CO_2_ to formate. However, it is important to consider that FDH has a higher affinity for the oxidation reaction of formate to CO_2_.^33^ Consequently, the accumulation of NAD^+^ in the medium after CO_2_ reduction could potentially enhance the formate oxidation reaction. Therefore, GlyDH assumes a pivotal role by catalyzing the NAD^+^ reduction back to NADH. Thus, by regenerating the cofactor, a favorable environment for CO_2_ reduction is re-established. Hence, different GlyDH concentrations were assessed to ascertain the optimal ratio, aiming to achieve maximal yields in the concurrent coproduction of formate and DHA.

On the other hand, in order to further study the multienzymatic system considering the gaseous proportion of CO_2_ most commonly found in the industry,^34^ a gas mixture containing 24% CO_2_ was employed as the substrate for FDH in the subsequent reactions (the rest was supplemented with nitrogen). At this CO_2_ proportion, the pH in the medium was maintained at around 7.1. Specifically, in the pH range of 7.0–7.4, the concentration of soluble CO_2_ ranges between 100–350 mg·L^–1^ with this gas mixture (Figure S2). Therefore, the viability of the multienzyme system simulating industrial and environmental conditions was assessed using this gas mixture.

As depicted in Figure 4, the production of both formate and DHA is directly proportional to the quantity of the GlyDH enzyme in the range of ratios from 1:1 to 1:6. Beyond the 1:5 ratio, the amount of DHA produced remains constant, while for formate, this behavior is observed from the 1:7 ratio onward. In this context, GlyDH effectively fulfills its role in regenerating the cofactor, enabling the continuous coproduction of formate and DHA.

It is crucial to emphasize the significant disparity in the quantities of formate and DHA produced across all of the ratios assessed. This gap becomes notably less pronounced as the concentration of GlyDH in the reaction mixture increases. Therefore, considering all of the points mentioned earlier, the ratio of 1:7 FDH/GlyDH was chosen as the optimal concentration for conducting this enzymatic multisynthesis. Within this ratio, the concentrations of 3.6 ± 0.12 and 5.0 ± 0.04 mM were achieved for formate and DHA, respectively, after 24 h of reaction. An experiment with this ratio of enzymes was conducted to examine the kinetics of this reaction over time.

**Figure 5:**
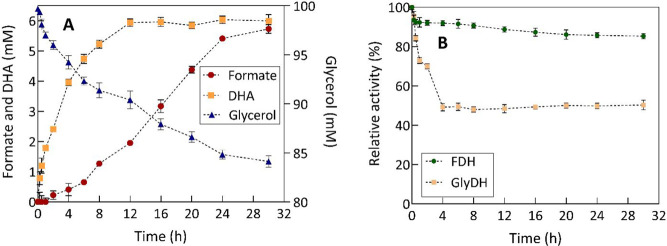
(A) Time course of formate and DHA coproduction by a multienzymatic system with NADH regeneration. (B) FDH and GlyDH stability enzymes over time in the reaction. The experiments were performed with 250 mM phosphate buffer as the reaction medium, 100 mM glycerol, a gas mixture with 24% CO_2_ (320 mg·L^–1^), and 1 mM NADH. A ratio of 1:7 FDH/GlyDH was employed (0.5 and 3.5 U·mL^–1^ FDH and GlyDH, respectively). The assays were performed at 30 °C, a reaction volume of 12 mL, initial pH 7.10 (following CO_2_ bubbling), with constant agitation at 450 rpm for 30 h. Relative activity was calculated with zero reaction time set at 100%.

The time course of the coproduction reaction of formate and DHA using the 1:7 FDH/GlyDH ratio is shown in Figure 5A. Initially, there is an exponential increase in DHA production during the first 12 h of the reaction. Subsequently, the DHA concentration remains constant until the end of the reaction, suggesting that the maximum DHA production has been achieved. It should be mentioned that DHA has a well-documented inhibitory effect on GlyDH. This can be observed from low concentrations of DHA (≈ 0.54 mM).^23^ The noncompetitive inhibitory mechanism of DHA is a result of GlyDH having an inhibitory binding site that is distinct from the active site, allowing DHA to bind to it. This interaction could lead to a decrease in catalytic activity.^23^ Therefore, GlyDH could be susceptible to inhibition by DHA under reactive conditions. A peculiarity of DHA in this reaction is its selective capacity to interact with the amino groups of aromatic amino acids, leading to the generation of brown pigments through the Maillard reaction^35^ (Figure S4).

On the other hand, formate production exhibits a gradual rate at the beginning of the reaction; however, a subsequent acceleration becomes evident, particularly upon reaching the maximum DHA concentration in the reaction. As previously mentioned, FDH from *Candida boidinii* has a greater affinity for the oxidation reaction of formate than for the reduction of CO_2_.^36^ However, several studies have successfully documented this reaction,^31,37,38^ as in the present case. In addition, the reaction was also assessed by using NAD^+^ as the initial cofactor. However, the concentrations obtained were lower (2.1 ± 0.14 mM formate and 3.4 ± 0.21 mM DHA) (Figure S5).

The change in the CO_2_ source (gas mixture with only 24% CO_2_) led to a notable enhancement in FDH activity compared to the previous reactions with pure CO_2_. The FDH residual activity was 85.3 ± 1.36% after 30 h of reaction (Figure 5B). Consequently, reducing the soluble CO_2_ concentration can extend the catalytic longevity of FDH. In the case of GlyDH, a pronounced catalytic inactivation was observed under these reaction conditions. During the initial 4 h of the reaction, there is a noticeable decrease in the GlyDH activity, potentially related to inhibition by DHA. This time frame of the reaction is characterized by a significant production of DHA. The residual activity was 50.3 ± 2.41% after 30 h of reaction, in contrast to a control sample under nonreactive conditions, where GlyDH retained 85.4 ± 1.22% of its activity.

An additional fact to take into consideration is that the DHA reduction reaction generally features lower *K*_m_ values for the GlyDH, compared to the oxidation of glycerol.^39^ This behavior is significantly influenced by the pH of the medium. The glycerol oxidation reaction is catalyzed more effectively at the highly basic pH levels (10–11). In contrast, the DHA reduction reaction is favored at more neutral pH levels (7.0–8.0).^40^ As mentioned earlier, in this reaction, the enzyme GlyDH operates below its optimal pH for glycerol oxidation (pH 9.0). However, the effective production of DHA has also been extensively documented under similar conditions to this reaction.^23^ Moreover, a high concentration of glycerol (100 mM) was employed in this reaction to shift the reaction in favor of the oxidation of this alcohol.

Despite the challenges encountered, the successful coproduction of both products was achieved. After 30 h of reaction, 5.7 ± 0.36 mM formate and 6 ± 0.14 mM DHA were produced. Consequently, prolonging the reaction time led to a reduction in disparity in the production of both molecules.

Besides, an imbalance between the glycerol consumed and the DHA produced was also evident. A control sample of glycerol incubated with CO_2_ displayed a progressive decrease in its concentration. In the literature, it has been described that the interaction between CO_2_ and glycerol can lead to the formation of glycerol carbonate.^41^ This compound is a product derived from the valorization of bioglycerol, and it is of significant interest due to its high solubility in water and low toxicity, among other properties.^42^ To address this imbalance, the quantity of glycerol 1,2-carbonate in the reaction was measured. The HPLC analysis revealed a concentration of 7.1 ± 0.27 mM at the end of the reaction. Similarly, a concentration of 3.9 ± 0.18 mM was found in the control sample under nonreactive conditions. Consequently, under these reaction conditions, a secondary reaction uncatalyzed between CO_2_ and glycerol can take place.

### Multienzymatic Platform Tested under Relevant Industrial Environments: Synthetic Gas Mixture Mimicking Real Iron and Steel Industry Off-Gases and Real Crude of Glycerol from Biodiesel Production

To assess the feasibility of implementing the multienzymatic system with actual industrial off-gases, an experiment involving a synthetic gas mixture mimicking the composition of blast furnace off-gas in the iron & steel industry^15^ was conducted. Furthermore, a crude glycerol waste containing 64% v/v glycerol, derived from biodiesel production, was incorporated as a sacrificial substrate. Notably, the inclusion of crude glycerol enhances the feasibility of this multienzymatic system since the majority of glycerol applications conventionally utilize pure glycerol to generate add-value molecules, rather than crude glycerol,^43^ mainly due to the high costs associated with its purification. A comparison of the appearance of pure and crude glycerol is found in the Supporting Information (Figure S5).

**Figure 6:**
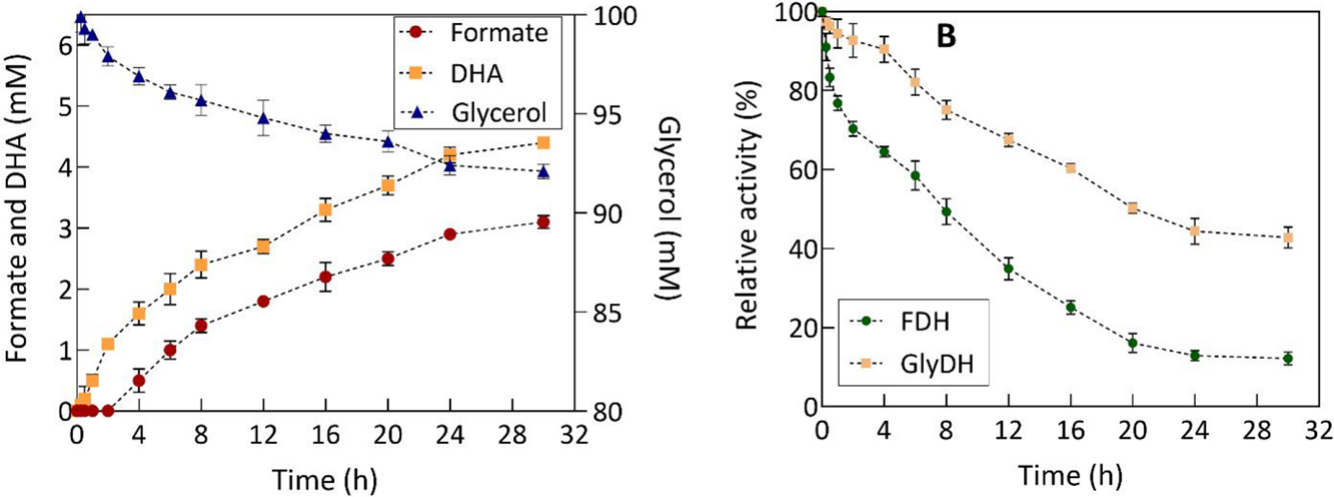
(A) Time course of formate and DHA coproduction by a multienzymatic system with NADH regeneration. (B) FDH and GlyDH stability enzymes over time in the reaction. The experiments were performed with 250 mM phosphate buffer as the reaction medium, 100 mM real crude glycerol, synthetic gas mixture mimicking real iron and steel industry off-gases (312 mg·L^–1^ of soluble CO_2_) and 1 mM NADH. A ratio of 1:7 FDH/GlyDH was employed (0.5 and 3.5 U·mL^–1^ FDH and GlyDH, respectively). The assay was performed at 30 °C, initial pH 7.15 (following CO_2_ bubbling), and constant agitation for 30 h. Relative activity was calculated with zero reaction time set at 100%.

The time course of the reaction using substrates under relevant industrial environment is illustrated in Figure 6A. The simultaneous production of formate and DHA was achieved successfully, obtaining concentrations of 3.1 ± 0.34 mM formate and 4.4 ± 0.11 mM DHA after 30 h of reaction. The production of both compounds is observed to proceed at a slower and more linear rate compared to the previous reaction. Similarly, a concentration of 1.8 ± 0.17 mM glycerol 1,2-carbonate was found at the end of the reaction, which elucidates the imbalance between the glycerol consumed and the DHA produced. This concentration is lower than the previous reaction (7.1 ± 0.27 mM), indicating a higher propensity of pure glycerol to generate this secondary product.

The productivity decrease with these substrates may be attributed to a more pronounced enzymatic inactivation under these reaction conditions. FDH retained only 12.2 ± 0.43% of its activity after 30 h of reaction (Figure 6B). In the case of GlyDH, a gradual inactivation is observed as the reaction progresses. However, the final residual activity of GlyDH ended up being slightly similar both in this reaction (44.8 ± 0.43%) and in the reaction with pure glycerol and the gas mixture with 24% CO_2_ (50.3 ± 2.41%).

**Figure 7:**
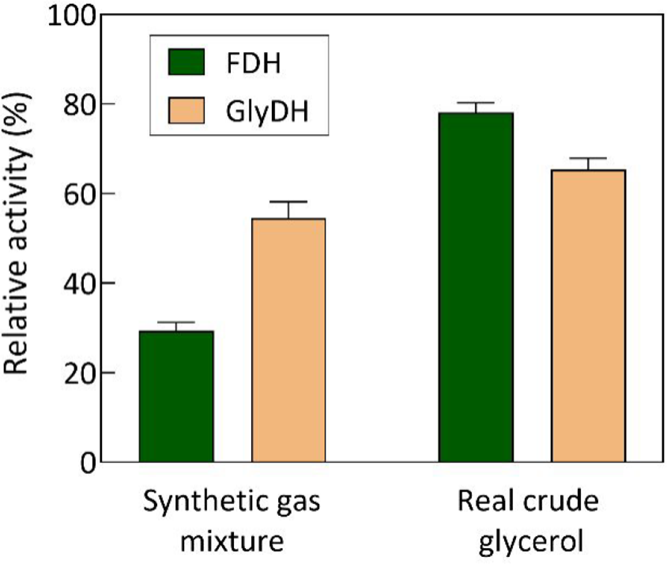
FDH and GlyDH stability when incubated in the synthetic gas mixture mimicking iron and steel industry off-gases (320 mg·L^–1^) and 100 mM real crude glycerol. The assays were performed for 30 h at a temperature of 30 °C with continuous stirring. Relative activity was calculated with zero reaction time set at 100%.

To assess this issue, both enzymes were incubated with each of the substrates involved in the reaction, allowing for the assessment of their enzymatic activity after a 30 h period. As depicted in Figure 7, both enzymes undergo varying degrees of inactivation upon contact with these substrates. In the case of the synthetic gas mixture, FDH residual activity was 29.3 ± 1.14%, while for GlyDH, it was 54.5 ± 0.91%. This inactivation may be associated with the presence of carbon monoxide (CO) gas, which can induce a degradative effect on these biocatalysts. In some enzymes of the dehydrogenase class, it has been observed that catalytic inactivation of approximately 20% occurs when they are exposed to 50 mM CO under standard temperature and pressure conditions.^44^ Furthermore, the composition of crude glycerol can also impact the catalytic performance of enzymes, primarily due to the high fat content that can result in the formation of surface-active species and a subsequent loss of enzymatic activity, in addition to other toxic substances, such as methanol.^45^

Finally, Table 1 presents the metrics for the parameters assessed in this multienzymatic system, facilitating a comparison between the reaction involving the gas mixture with 24% CO_2_ and glycerol and the reaction with the synthetic gas mixture and real crude glycerol. As observed, the first reaction resulted in higher yields, and a notably high percentage of CO_2_ conversion was achieved (92.3%). In contrast, the reaction with a synthetic gas mixture yielded a 50.8% CO_2_ conversion. The conversion of glycerol in both reactions was relatively lower, primarily due to the large amount of glycerol used (100 mM). Regarding space–time yield (STY), this platform exhibits reasonable productivity for the synthesis of formate (8.6 mg L^–1^ h^–1^) and DHA (17.9 mg L^–1^ h^–1^). The results of Table 1 were compared with those of previous reports (Table S1). In the case of enzymatic formate synthesis, most studies use FDH immobilized on various nanomaterials.^30,46,47^ In these studies, lower concentrations of formate are reported; however, the yields are much higher due to a shorter reaction time employed. Additionally, immobilized multienzyme systems have been widely adapted for the synthesis of this compound, using enzymes such as carbonic anhydrase to enhance CO_2_ solubility and glucose dehydrogenase and glutamate dehydrogenase for cofactor regeneration.^48,49^ The majority report lower formate concentrations than the present study but with significantly higher yields. In the case of DHA, many reports also feature GlyDH immobilized on various nanomaterials,^25,50^ as well as in multienzyme systems (both immobilized and free) with NADH oxidase (NOX)^23,51^ and xylose reductase.^52^ These multienzymatic systems show yields similar to those obtained in this study.

**Table 1:** Performance Parameters of the Multienzyme Synthesis Process for the Production of Formate and DHA with NADH Regeneration by Contrasting the Reaction with a Gas Mixture with 24% CO_2_ + Glycerol and the Reaction with the Synthetic Gas Mixture + Real Crude Glycerol

substrates	product	concentration (mM)	conversion (%)	STY (mg L^–1^ h^–1^)	TTN		
					(mol formate/mol NADH ^–1^)	(mol formate/mol FDH ^–1^)	(mol DHA/mol GlyDH ^–1^)
24% CO_2_^a^ + glycerol	formate	5.73 ± 0.32	92.3^b^	8.6	5.7	11,191	3380
	DHA	5.98 ± 0.14	15.6	17.9			
synthetic gas mixture + crude glycerol	formate	3.12 ± 0.34	50.8^c^	4.7	3.1	6094	2499
	DHA	4.42 ± 0.11	8.4	13.3			

a Simulated CO_2_: 24% CO_2_ and 76% N_2_. Synthetic gas mixture: 24.5% CO_2_, 1.2% O_2_, 3.8% H_2_, 23.9% CO, and 46.6% N_2_.

b Based on 7.3 mM CO_2_ soluble (medium presaturated with CO_2_ corresponds to 85% of the total volume).

c Based on 7.1 mM CO_2_ soluble (medium presaturated with CO_2_ corresponds to 80% of the total volume).

Cocuzza reported a similar multienzyme system to the one in the present study but with FDH and GlyDH enzymes co-immobilized in modified natural zeolite. After 2 h, a concentration of 3 mM formic acid was obtained; however, the synthesis of DHA was not addressed in this study.^53^ In most of these studies, the TTN values based on the cofactor were similar or lower than those obtained in this study, demonstrating in this way the system’s viability in simultaneously producing two molecules from a low concentration of NADH cofactor.

### Sustainable Formate Recovery from Bioconversion Using 2-Methyltetrahydrofuran

To assess the feasibility of the system, formic acid was purified using liquid–liquid extraction with 2-methyltetrahydrofuran (2-MTHF), a biorenewable solvent derived from lignocellulosic biomass. For the reaction with the gas mixture containing 24% CO_2_ and glycerol, 90.5% of the produced formate (in the form of formic acid) was extracted into 2-MTHF. In the case of the reaction with the synthetic gas mixture and crude glycerol, an extraction efficiency of 85.2% was achieved. This resulted in the recovery of 2.83 and 1.47 mg of formic acid, respectively (Table S2, SI). Regarding the remaining compounds of the multienzymatic system, the largest amount of them was retained in the aqueous phase following the liquid–liquid extraction with 2-MTHF. At least 93.4% of the total DHA produced in both reactions was retained in the aqueous phase (Table S3, SI).

Subsequent distillation to separate formic acid from 2-MTHF yielded lower recoveries of 60.1% and 55.8%, respectively, resulting in a final yield of 1.88 and 0.9 mg of formic acid. This reduced efficiency can be attributed to the formation of an azeotrope between formic acid and 2-MTHF, as previously reported.^54^ Despite this limitation, 2-MTHF remains a promising and cost-effective solvent for formic acid extraction, particularly from dilute stream of formic acid.^54^

Regarding DHA recovery, adsorption resins can be applied. This approach has been successfully employed in other biocatalytic processes for DHA production.^55^ Further investigation will focus on exploring this approach, along with the intensification of the multienzymatic process (e.g., immobilized enzymes) and integration with downstream processes.

## Conclusions

In summary, the reduction of CO_2_ to formate by the FDH enzyme was assessed in a multienzymatic system aimed at valorizing low-cost industrial wastes and transforming them into high-value compounds. The successful in situ NADH regeneration was achieved by incorporating another industrial waste, glycerol. This approach yielded two high-value molecules, resulting in 5.7 ± 0.32 mM formate and 6 ± 0.14 mM DHA using a gas mixture with 24% CO_2_ and glycerol as substrates. Furthermore, the reaction was effectively conducted by the substrates under relevant industrial conditions, such as a gas mixture mimicking CO_2_ from the blast furnace off-gas in the iron and steel industry and crude glycerol from the biodiesel production, obtaining 3.1 ± 0.34 mM formate and 4.4 ± 0.11 mM DHA. Furthermore, formic acid was successfully isolated through liquid–liquid extraction with 2-MTHF, achieving extraction efficiencies of 90.5 and 85.2% from the respective reactions. However, these findings may pave the way for further exploration of multienzymatic systems on a large scale, employing immobilized biocatalysts to maximize the yields and the robustness of the enzymes. Hence, the coproduction of two industrially relevant molecules from waste materials occurred within a robust, environmentally friendly, cost-effective, and easily manageable multienzymatic system, all under mild operating conditions.

## Data Availability

The data supporting this study are openly available in the CORA repository at https://doi.org/10.34810/data1749.

## Supporting Information Available

Production and purification of FDH and GlyDH enzymes; NADH stability test; correlation pH vs CO_2_ solubility; maillard reaction; reaction with NAD^+^; pure vs crude glycerol; HPLC chromatograms and a comparison of enzymatic synthesis of formate and DHA between previous reports and this study; and purification of formic acid and the composition of crude glycerol (DOCX)
