# Supplementary material for: Multienzymatic Platform for Coupling a CCU Strategy to Waste Valorization: CO2 from the Iron and Steel Industry and Crude Glycerol from Biodiesel Production
Source: ACS Sustain Chem Eng. 2025 Jan 17;13(4):1440–9. doi: 10.1021/acssuschemeng.4c04908 (PMC11795641; doi:10.1021/acssuschemeng.4c04908)
Supplement: Supplementary file 1 — sc4c04908_si_001.docx [file sc4c04908_si_001.docx]

**Supporting Information (SI).**

Multienzymatic Platform for Coupling a CCU Strategy to Waste Valorization: CO_2_ from Iron and Steel Industry and Crude Glycerol from Biodiesel Production.

Sady Roberto Rodriguez†, Gregorio Álvaro†, Marina Guillén† & Oscar Romero†*.

†Bioprocess Engineering and Applied Biocatalysis Group, Department of Chemical, Biological and Environmental Engineering, Universitat Autònoma de Barcelona, 08193 Bellaterra, Spain.

Number of pages: **13**

Figures: **9**

Table: **4**

**Materials and Methods.**

***Materials***

All reagents were purchased from Sigma Aldrich® (St. Louis, MO, USA) and PanReac Quimica S.L.U. (Barcelona, Spain). All samples and buffers were prepared in Milli Q water (18.2 MΩ cm, Merck Millipore, USA). Mini-PROTEAN® TGX™ Precast Protein Gels and Precision Plus Protein™ All Blue Standards were purchased from BIO-RAD (Madrid, Spain). InstantBlue® Coomassie Protein Stain was purchased from Abcam® (Cambridge, UK). The full composition of crude glycerol as follow: Glycerol 64%, Water 12%, MONG (Matter Organic Non-Glycerol) 13%, Ashes 11.5% and Methanol 0.04%. This crude glycerol has a pH of 5.5.

***Enzymes production and purification.***

FDH from *Candida boidinii* and GlyDH from *Geobacillus stearothermophilus* were expressed in recombinant *Escherichia coli* BL21 (DE3) strains transformed with pet26b(+), kanamycin resistance and with His-tag. Briefly, a pre-inoculum was prepared by adding 100 µL of glycerol stocks to 25 mL of defined media (composition described by Vidal *et al*.^1^ ) and incubated overnight at 37 ^°^C with 150 rpm agitation. Inoculum cultures were prepared twice with 200 mL of defined media, inoculated with pre-inoculum at an initial OD_600_ of 0.2 and incubated at 37 ^0^C with 150 rpm agitation. When the inoculum reached an OD_600_ of 1.0, they were transferred to 2.5 L of defined media prepared in a 5 L bioreactor Applikon®. An initial batch-phase was performed at 30 ^°^C, pH 7.0 and pO_2_ 30% adjusting the agitation between 450 and 1150 rpm and supplying air and/or pure oxygen at a flow of 1.5 L min^-1^ according to the biomass requirements. A 1:10 antifoam solution was used throughout the process. The pH was controlled with a NH_4_OH 15% v/v solution. When the glucose was completely consumed, the fed-batch phase was initiated with a preprogrammed exponential addition of feeding medium (composition defined by Vidal *et al*.^1^). The induction phase was started when an OD_600_ between 100 - 120 was reached, by the addition of β-D-1-thiogalactopyranoside (IPTG) at a 0.1 mM final concentration. The induction phase was carried out for 4 hours.

The biomass obtained was centrifuged at 10,000 rpm for 15 minutes at 4 °C. *E. coli* cells expressing FDH enzyme were resuspended in phosphate buffer 100 mM and NaCl 100 mM (pH 7.0) and *E. coli* cells expressing GlyDH enzyme were resuspended in phosphate buffer 10 mM (pH 7.0). Cells suspensions were disrupted using a cell disruptor (Constant System Ltd.) followed by a centrifugation of the resulting cell lysate (10,000 rpm for 45 minutes at 4 °C).

Cell lysates were purified using an AKTA Pure 150 FPLC system (GE Healthcare®). Affinity chromatography was used for the purification of FDH enzyme, which consists of a column packed with agarose functionalized with nickel ions (Ni^+2^). The column was initially equilibrated with phosphate buffer 100 mM and NaCl 100 mM (pH 7.0). This same buffer was used to load the sample and then wash the column before elution. Unbounded proteins were eluted during this step. FDH was eluted from the column by applying phosphate 100 mM, NaCl 100 mM and imidazole 300 mM buffer (pH 7.0). In the case of GlyDH, an ion exchange method was used with a column packed with diaminoethyl-agarose. Equilibration, sample loading and washing were carried out with 10 mM phosphate buffer (pH 7.0). Unbound material was washed out of the column using this buffer. GlyDH enzyme was eluted with a linear gradient using phosphate 100 mM + NaCl 300 mM buffer (pH 7.0). All the proteins were detected under 280 nm UV light. Catalytic activity and protein concentration were measured for each fraction obtained. Protein concentration was measured by Bradford’s method^2^ , using Bovine serum albumin (BSA) as a standard. The samples containing FDH and GlyDH were dialyzed on a cellulose membrane using phosphate buffer 100 mM (pH 7.5) for 1 h, overnight, and then for 1 h more.

***SDS-PAGE electrophoresis.***

The molecular weights were estimated by the electrophoretic mobility of the proteins in a Sodium Dodecyl Sulfate Polyacrylamide Gel (SDS-PAGE). Samples were prepared at a concentration of 0.5 mg mL and incubated in the loading buffer (Laemmli buffer 4x + β- mercaptoethanol 10:1) for 10 minutes at 95 ^°^C. Subsequently, 15 µL of sample and 7 µL of standard proteins were loaded into the gel. Electrophoresis was performed for 80 minutes at 120 V. The gel was stained with Coomassie InstantBlue® and washed with water until blue bands appeared. The gel was analyzed using the Image Lab software from BIO-RAD.

***NADH quantification and stability test.***

NADH stability was evaluated in a pH range from 6.0 to 9.0. Different Britton-Robinson buffer solutions were prepared and adjusted to the pH under study. The experimental conditions involved a 1 mM NADH concentration, temperature of 30 °C and for undefined duration. NADH was quantified by UV/visible spectrophotometry at 340 nm using the SPECTROstar microplate reader. The relative percentages were calculated considering zero-time samples as 100% NADH initial concentration. Triplicate assays were performed.


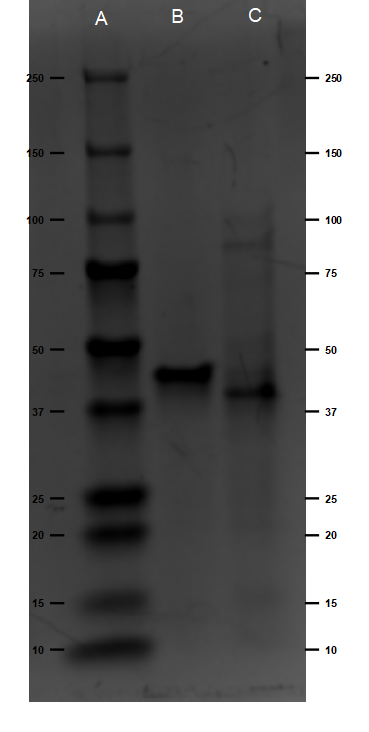


**Figure S1**. SDS-PAGE of purified Formate dehydrogenase (FDH) and Glycerol dehydrogenase (GlyDH) enzymes. (A) Standard proteins. (B) FDH enzyme (41 kDa^3^ ). (C) GlyDH enzyme (39.5 kDa^4^ )


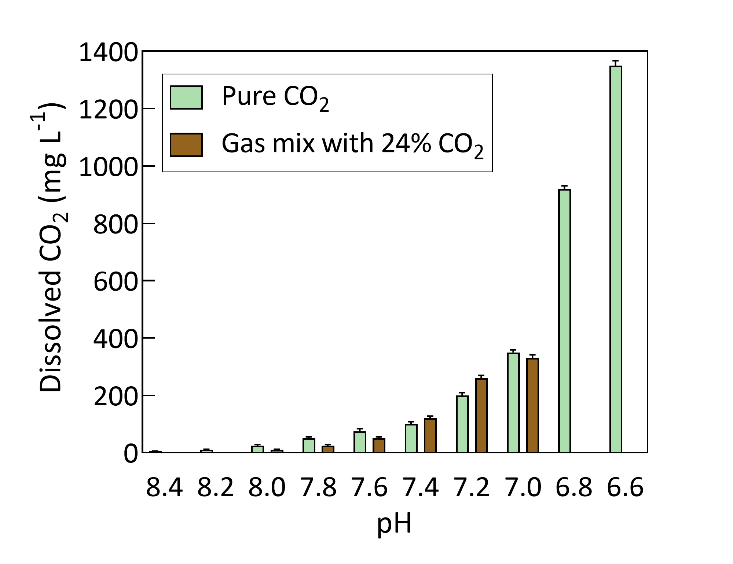


**Figure S2.** Correlation between the pH and the CO_2_ solubility in the reaction medium, evaluating pure CO_2_ and a gas mixture with 24% CO_2_ and 76% N_2._


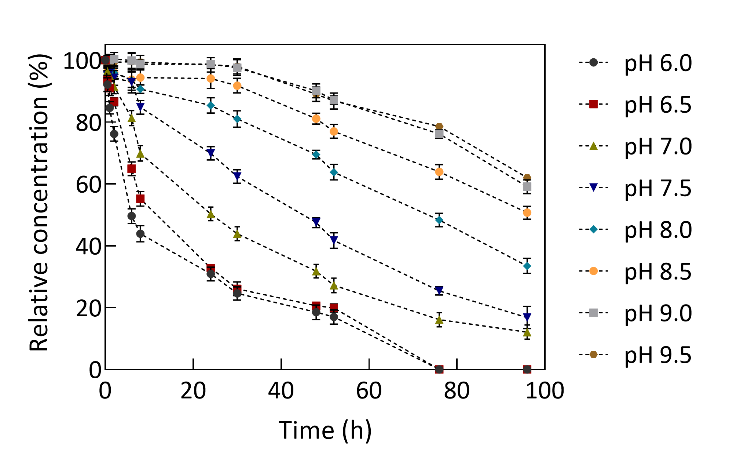


**Figure S3**. Stability study of NADH cofactor at different pH levels. The assay was performed in a pH range from 6.0 to 9.5 using phosphate buffer 250 mM at 30 °C. Zero incubation time was considered as 100% NADH concentration.


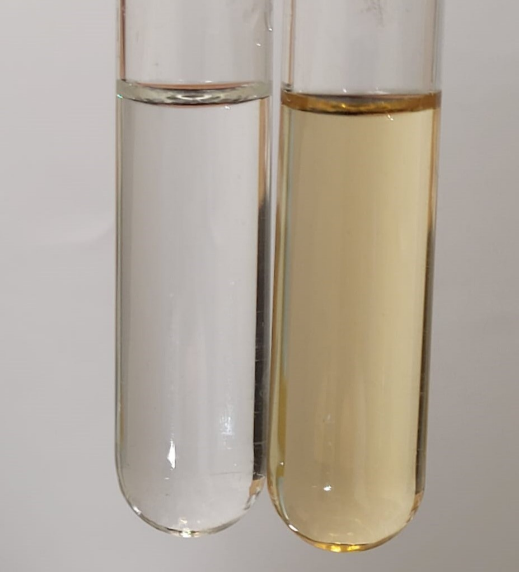


**Figure S4.** Comparison of the coloration in the reaction medium resulting from the interaction between DHA and the amino groups of the enzymes through the Maillard reaction. Left: Initial coloration (zero reaction time). Right: Coloration after 30 hours of reaction.


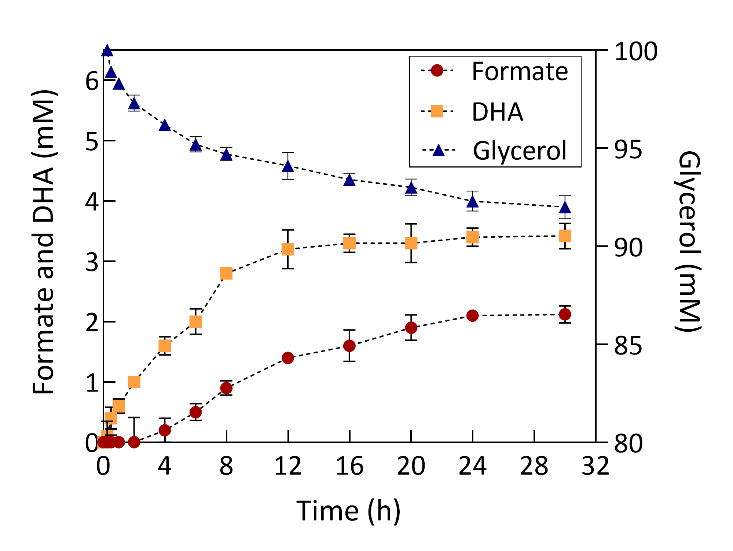


**Figure S5.** Time course of formate and DHA co-production by a multienzymatic system with NAD^+^ regeneration. The experiments were performed with phosphate buffer 250 mM as the reaction medium, glycerol 100 mM, gas mixture with 24% CO_2_ (318 mg L^-1^) and NAD^+^ 1 mM. A ratio of 1:7 FDH:GlyDH was employed (0.5 and 3.5 U mL^-1^ of FDH and GlyDH, respectively). The assay was performed at 30 °C, initial pH 7.12 (following CO_2_ bubbling) and constant agitation for 30 hours. Relative activity was calculated with zero reaction time set at 100%.


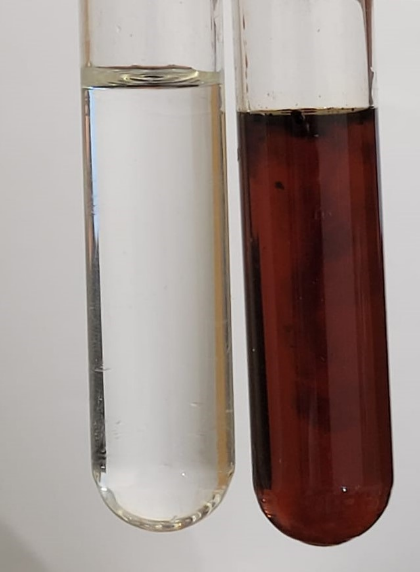


**Figure S6.** Comparison between pure glycerol (left) and crude glycerol (right).


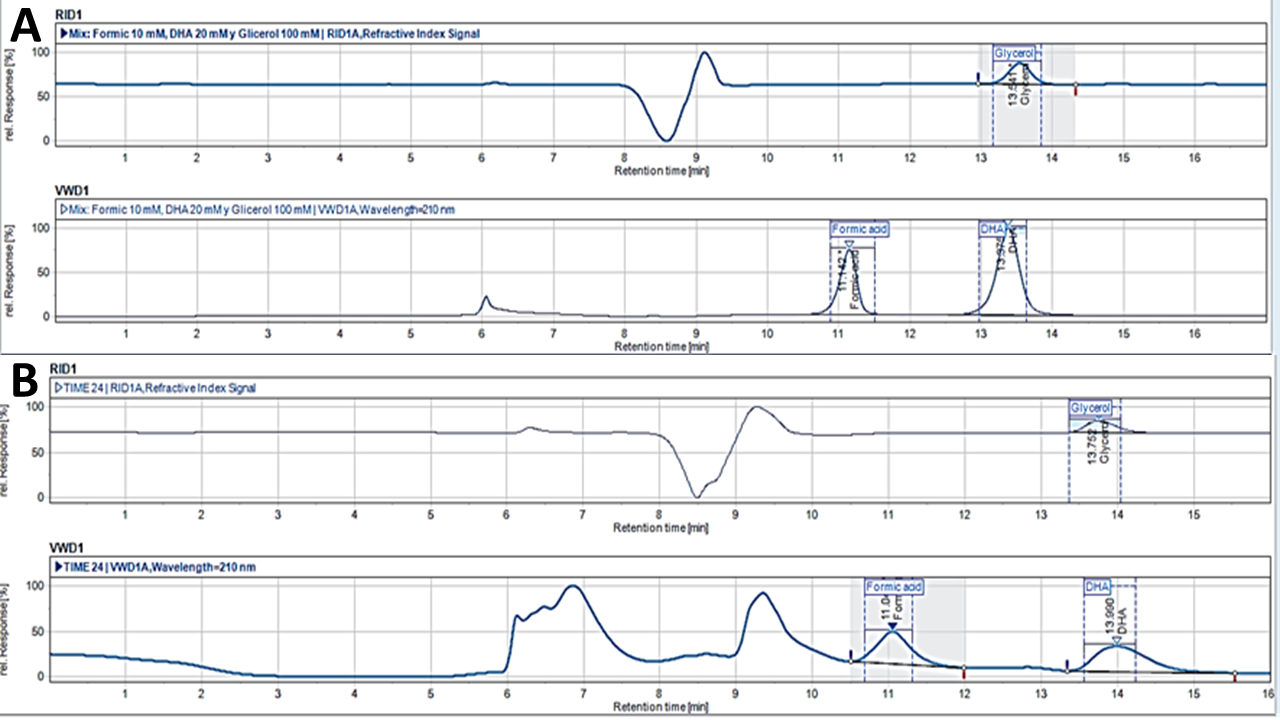


**Figure S7.** HPLC chromatograms. (A) Standards of sodium formate 10 mM (RT: 11.1 min), DHA 20 mM (RT: 13.4 min) and glycerol 100 mM (RT: 13.5 min). (B) 24-hours sample of the co-production of formic acid and DHA using the gas mixture with 24% CO_2_ and pure glycerol. RT formate: 11 min, DHA RT: 13.9 min and glycerol RT: 13.8 min. Analysis conditions: Column: IC-Sep COREGEL 87H3, mobile phase: H_2_SO_4_ 0.5 mM: Acetonitrile (65:35), flow rate: 0.6 mL min^-1^, injection volume: 20 µL, column temperature: 30°C, UV/Visible detector (WWD1): 210 nm and refractive index detector (RID) at 30°C.


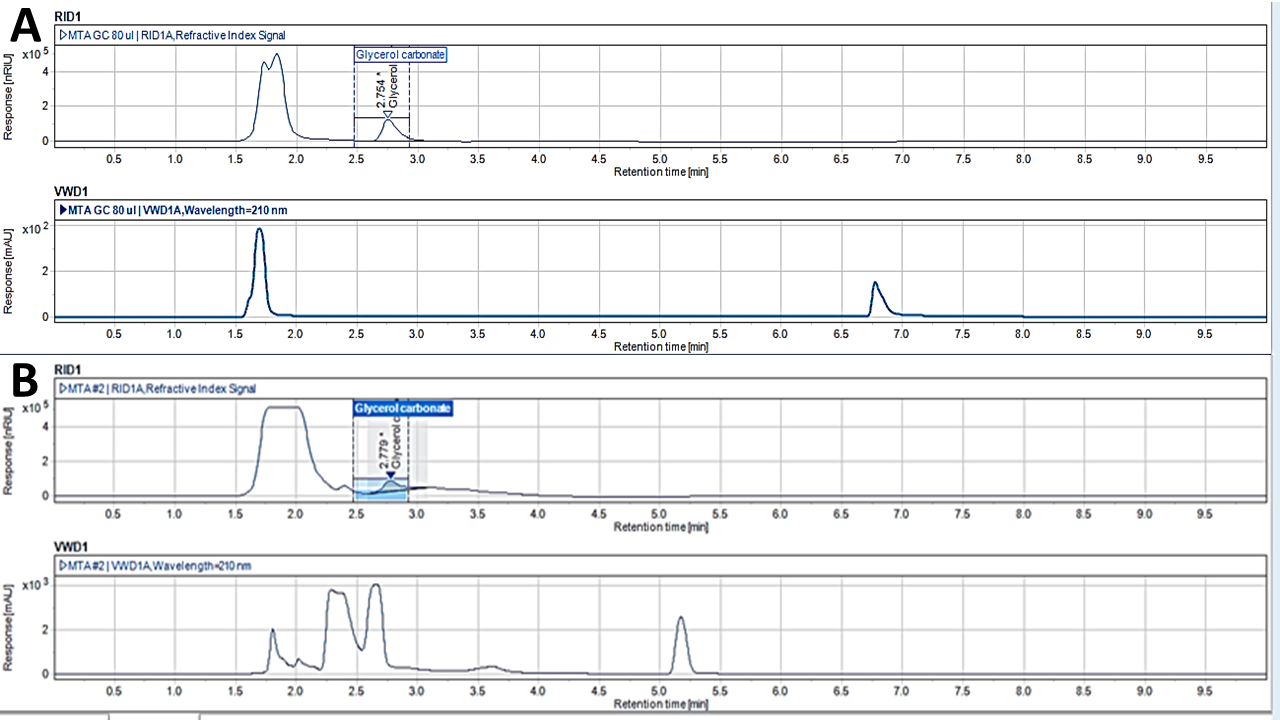


**Figure S8.** HPLC chromatograms of glycerol 1,2- carbonate quantification. (A) Standard of glycerol 1,2 – carbonate 20 mM (RT: 2.8 min) (B) 24-hour sample of the co-production of formic acid and DHA using the gas mixture with 24% CO_2_ and pure glycerol. RT glycerol 1,2 – carbonate: 2.8 min. Analysis conditions: Column: C-18 CORTECS (90Å, 2.7 µm, 4.6 mm x 150 mm and 250 mm), mobile phase: H_2_SO_4_ 0.004N, flow rate: 0.8 mL min^-1^, injection volume: 100 µL, column temperature and refractive index detector (RID) at 35°C.


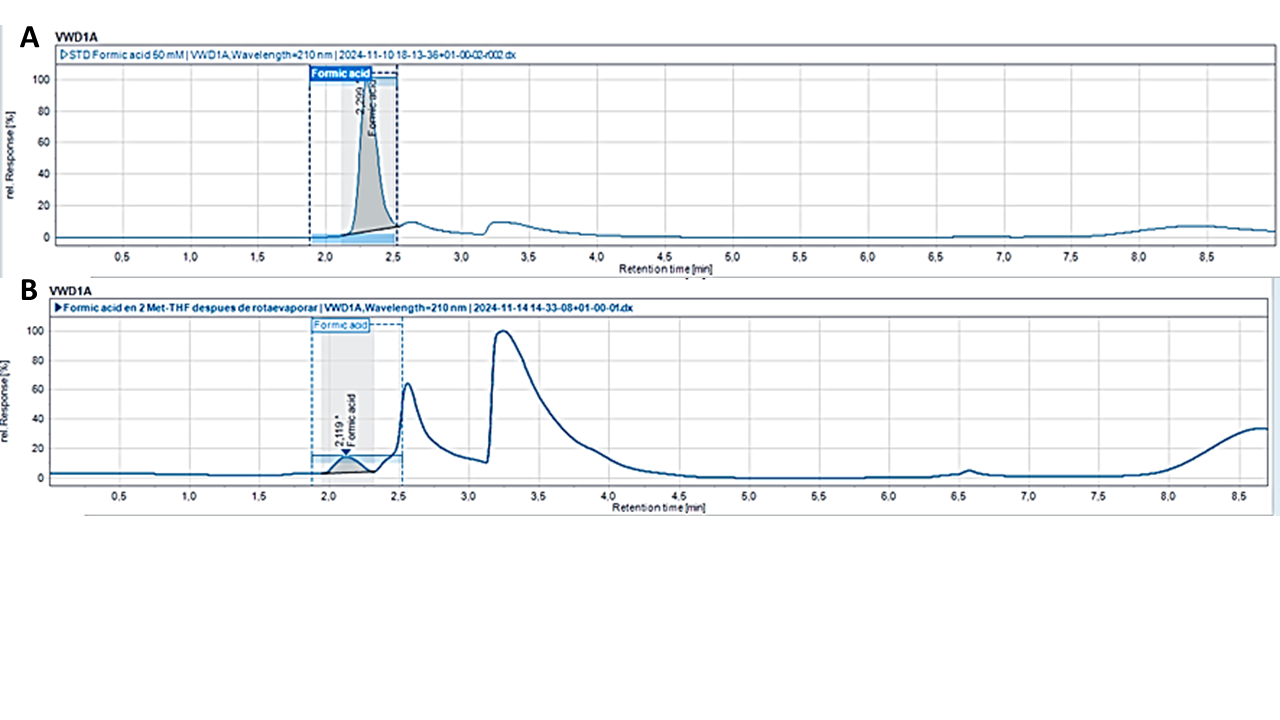


**Figure S9.** HPLC chromatograms of formic acid in 2-methyl tetrahydrofuran (2-MTHF) quantification. (A) Standard of formic acid 50 mM (RT: 2.2 min) (B) Sample of formic acid extraction in 2-MTHF (RT: 2.1 min). Analysis conditions: Column: C-18 CORTECS (90Å, 2.7 µm, 4.6 mm x 150 mm and 250 mm), mobile phase: H_2_SO_4_ 0.5 mM: Acetonitrile (65:35), flow rate: 0.6 mL min-1, injection volume: 20 µL, column temperature: 30°C and UV/Visible detector (WWD1): 210 nm.

**Table S1.** Comparison of metrics on the enzymatic synthesis of formate and DHA between previous reports and this study.

| Biocatalyst | Product | Concentration (mM) | Conversion (%) | STY (mg L^-1^ h^-1^) | TTN (mol product / mol cofactor ^-1^) | Reaction time (h) | Reference |
| --- | --- | --- | --- | --- | --- | --- | --- |
| Free FDH | Formic acid | 4 | NR | 92 | NR | 2 | ^5^ |
| Free FDH | Formic acid | 1.8 | 36 | 165 | NR | 0.5 | ^6^ |
| FDH immobilized on MWCNT/Ni/NiO nanotubes |  |  |  |  |  |  |  |
| FDH immobilized on mesoporous silica | Formic acid | 0.08 | 0.8* | 1.8 | NR | 2 | ^7^ |
| Multienzymatic system with FDH and glucose dehydrogenase | Formate / Formic acid | 0.9 | 3.2 | 4.1 | 1.8 | 10 | ^8^ |
| Multienzymatic system with carbonic anhydrase, glutamate dehydrogenase and FDH co-encapsulated in ZIF-8. | Formate / Formic acid | 13 | NR | 598 | NR | 1 | ^9^ |
| Multienzymatic system with carbonic anhydrase, glutamate dehydrogenase and FDH immobilized in metal-organic frameworks (MOFs) | Formate / Formic acid | 5 | 89 | 38 | 2.3 | 6 | ^10^ |
| Multienzymatic system with free GlyDH and xylose reductase | DHA | 7.2 | NR | 32.4 | 72 | 20 | ^11^ |
| GlyDH immobilized in magnetically mesoporous silica | DHA | 0.189 | 85** | 0.35 | NR | 48 | ^12^ |
| GlyDH immobilized on superparamagnetic silica nanoparticles | DHA | 3.5 | 30.4** | 315 | NR | 1 | ^13^ |
|  |  |  |  |  |  |  |  |
| **Table S1.** (continuation) | | | | | | | |
| Multienzymatic system with GlyDH and NADH oxidase (NOX) co-immobilized on epoxy-functionalized magnetic nanoparticles | DHA | 3.5 | 3.5 | 13.1 | 0.018 | 24 | ^14^ |
| Multienzymatic system with free GlyDH and NOX | DHA | 1 | 5 | 11.3 | NR | 8 | ^15^ |
| Multienzymatic system with FDH and GlyDH co-immobilized on modified natural zeolite | Formic acid | 3 | NR | 69 | NR | 2 | ^16^ |
|  | DHA | NR |  | NR |  |  |  |
| Multienzymatic system with free FDH and GlyDH | Formate | 5.7 | 92.3 | 8.6 | 5.7 | 30 | This study |
|  | DHA | 6 | 15.6 | 17.9 | 6 |  |  |

NR: Not reported*Based on NADH conversion

**Based on NAD^+^ conversion

**Table S2.** Material balance of the downstream process by liquid-liquid extraction with 2-methyltetrahydrofuran (2-MTHF) and followed by solvent recovery through rotary evaporation for the separation of formic acid produced in the two evaluated reactions.

| Downstream process | Reaction with 24% CO_2_ + glycerol | | | | | Reaction with synthetic gas mixture + crude glycerol | | | | |
| --- | --- | --- | --- | --- | --- | --- | --- | --- | --- | --- |
|  | Concentration (mM) | Volume (mL) | mmol formate | mg formate | Recovery (%) | Concentration (mM) | Volume (mL) | mmol formate | mg formate | Recovery % |
| Reaction medium | 5.66 | 12 | 0.068 | 3.13 | --- | 3.12 | 12 | 0.037 | 1.72 | --- |
| Extraction with 2-MTHF | 1.28 | 48 | 0.062 | 2.83 | 90.5 | 0.66 | 48 | 0.032 | 1.47 | 85.2 |
| Rotary evaporation | 7.99 | 5 | 0.041 | 1.88 | 60.1 | 4.18 | 5 | 0.021 | 0.96 | 55.8 |

**Table S3.** Material balance of all compounds of the multienzymatic system after liquid-liquid extraction with 2-methyltetrahydrofuran (2-MTHF) for the separation of formic acid produced in the two evaluated reactions*

| Sample | Reaction with 24% CO_2_ + glycerol | | | | Reaction with synthetic gas mixture + crude glycerol | | | |  |
| --- | --- | --- | --- | --- | --- | --- | --- | --- | --- |
|  | Formate | DHA | Glycerol | Glycerol 1,2 – carbonate | Formate | DHA | Glycerol | Glycerol 1,2 – carbonate | |
| Reaction medium | 3.13 | 5.20 | 93.74 | 9.83 | 1.72 | 4.01 | 102.23 | 2.52 | |
| Aqueous phase after formic acid extraction with 2- MTHF | 0.29 | 4.86 | 84.36 | 7.82 | 0.23 | 3.76 | 90.94 | 2.18 | |

*Results expressed in mass units (milligrams)

**Table S4.** Crude glycerol data sheet provided by Ecomotion Biodiesel S.A.

| **Analysis** | **Result (%)** |
| --- | --- |
| Glycerol | 64 |
| Water | 12 |
| MONG* | 13 |
| Ashes | 11.5 |
| Methanol | 0.04 |
| pH | 5.5 |

********MONG: Matter Organic Non-Glycerol.*

**REFERENCES**

1. Vidal, L., Pinsach, J., Striedner, G., Caminal, G. & Ferrer, P. Development of an antibiotic-free plasmid selection system based on glycine auxotrophy for recombinant protein overproduction in Escherichia coli. *J. Biotechnol.* **134**, 127–136 (2008).

2. Bradford, M. M. A rapid and sensitive method for the quantitation of microgram quantities of protein utilizing the principle of protein-dye binding. *Anal. Biochem.* **72**, 248–254 (1976).

3. Labrou, N. E. Improved purification of Candida boidinii formate dehydrogenase. *Bioseparation* **9**, 99–104 (2000).

4. Ruzheinikov, S. N. *et al.* Glycerol Dehydrogenase: Structure, Specificity, and Mechanism of a Family III Polyol Dehydrogenase. *Structure* **9**, 789–802 (2001).

5. Kim, Y.-K., Lee, S.-Y. & Oh, B.-K. Enhancement of formic acid production from CO_2_ in formate dehydrogenase reaction using nanoparticles. *RSC Adv.* **6**, 109978–109982 (2016).

6. Tülek, A. *et al.* Sustainable production of formic acid from CO_2_ by a novel immobilized mutant formate dehydrogenase. *Sep. Purif. Technol.* **309**, 123090 (2023).

7. Pietricola, G. *et al.* Synthesis and characterization of ordered mesoporous silicas for the immobilization of formate dehydrogenase (FDH). *Int. J. Biol. Macromol.* **177**, 261–270 (2021).

8. Yu, X. *et al.* Synthesis of Formate from CO_2_ Gas Catalyzed by an O_2_-Tolerant NAD-Dependent Formate Dehydrogenase and Glucose Dehydrogenase. *Biochemistry* **58**, 1861–1868 (2019).

9. Ren, S. *et al.* Co-immobilization multienzyme nanoreactor with co-factor regeneration for conversion of CO_2_. *Int. J. Biol. Macromol.* **155**, 110–118 (2020).

10. Li, Y., Wen, L., Tan, T. & Lv, Y. Sequential Co-immobilization of Enzymes in Metal-Organic Frameworks for Efficient Biocatalytic Conversion of Adsorbed CO_2_ to Formate. *Front. Bioeng. Biotechnol.* **7**, 1–13 (2019).

11. Zhang, Y. *et al.* Simultaneous production of 1,3-dihydroxyacetone and xylitol from glycerol and xylose using a nanoparticle-supported multi-enzyme system with in situ cofactor regeneration. *Bioresour. Technol.* **102**, 1837–1843 (2011).

12. Kumar, G. S. *et al.* Stabilized glycerol dehydrogenase for the conversion of glycerol to dihydroxyacetone. *Chem. Eng. J.* **276**, 283–288 (2015).

13. Zheng, M. & Zhang, S. Immobilization of glycerol dehydrogenase on magnetic silica nanoparticles for conversion of glycerol to value-added 1 , 3-dihydroxyacetone. *Biocatal. Biotransformation* **29**, 278–287 (2011).

14. Zhuang, M. *et al.* Immobilization of glycerol dehydrogenase and NADH oxidase for enzymatic synthesis of 1 , 3-dihydroxyacetone with in situ cofactor regeneration. *J. Chem. Technol. Biotechnol.* **93**, 2351–2358 (2018).

15. Rocha-Martin, J., Acosta, A., Berenguer, J., Guisan, J. M. & Lopez-Gallego, F. Selective oxidation of glycerol to 1,3-dihydroxyacetone by covalently immobilized glycerol dehydrogenases with higher stability and lower product inhibition. *Bioresour. Technol.* **170**, 445–453 (2014).

16. Cocuzza, C. *et al.* Simultaneous CO_2_ reduction and NADH regeneration using formate and glycerol dehydrogenase enzymes co-immobilized on modified natural zeolite. *RSC Adv.* **12**, 31142–31155 (2022).
